# Methylammonium-free co-evaporated perovskite absorbers with high radiation and UV tolerance: an option for in-space manufacturing of space-PV?†

**DOI:** 10.1039/d3ra03846g

**Published:** 2023-07-12

**Authors:** Felix Lang, Yu-Hsien Chiang, Kyle Frohna, Sercan Ozen, Heinz C. Neitzert, Andrea Denker, Martin Stolterfoht, Samuel D. Stranks

**Affiliations:** a Department of Physics, Cavendish Laboratory, University of Cambridge JJ Thomson Avenue CB3 0HE Cambridge UK lang1@uni-potsdam.de sds65@cam.ac.uk; b Institute of Physics and Astronomy, University of Potsdam Karl-Liebknecht-Str. 24–25 Potsdam-Golm D-14476 Germany; c Department of Industrial Engineering (DIIn), Salerno University Fisciano SA Italy; d Helmholtz-Zentrum Berlin für Materialien und Energie GmbH, Protonen für die Therapie Hahn-Meitner Platz 1 14109 Berlin Germany; e Beuth Hochschule für Technik Berlin, Fachbereich II – Mathematik – Physik – Chemie Luxemburgerstr. 10 D-13353 Berlin Germany; f Department of Chemical Engineering & Biotechnology, University of Cambridge Philippa Fawcett Drive CB3 0AS Cambridge UK

## Abstract

With a remarkable tolerance to high-energetic radiation and potential high power-to-weight ratios, halide perovskite-based solar cells are interesting for future space PV applications. In this work, we fabricate and test methylammonium-free, co-evaporated FA_0.7_Cs_0.3_Pb(I_0.9_Br_0.1_)_3_ perovskite solar cells that could potentially be fabricated in space or on the Moon by physical vapor deposition, making use of the available vacuum present. The absence of methylammonium hereby increased the UV-light stability significantly, an important factor considering the increased UV proportion in the extra-terrestrial solar spectrum. We then tested their radiation tolerance under high energetic proton irradiation and found that the PCE degraded to 0.79 of its initial value due to coloring of the glass substrate, a typical problem that often complicates analysis. To disentangle damage mechanisms and to assess whether the perovskite degraded, we employ injection-current-dependent electroluminescence (EL) and intensity-dependent *V*_OC_ measurements to derive pseudo-*JV* curves that are independent of parasitic effects. This way we identify a high radiation tolerance with 0.96 of the initial PCE remaining after 1 × 10^13^ p^+^ cm^−2^ which is beyond today's space material systems (<0.8) and on par with those of previously tested solution-processed perovskite solar cells. Together our results render co-evaporated perovskites as highly interesting candidates for future space manufacturing, while the pseudo-*JV* methodology presents an important tool to disentangle parasitic effects.

## Abstract 2

In this work, we fabricate & test MA-free co-evaporated perovskite solar cells that could potentially be fabricated in space. Pseudo-*JV* characteristics & electroluminescence hereby help to distinguish radiation damage from parasitic effects.

## Graphical abstract

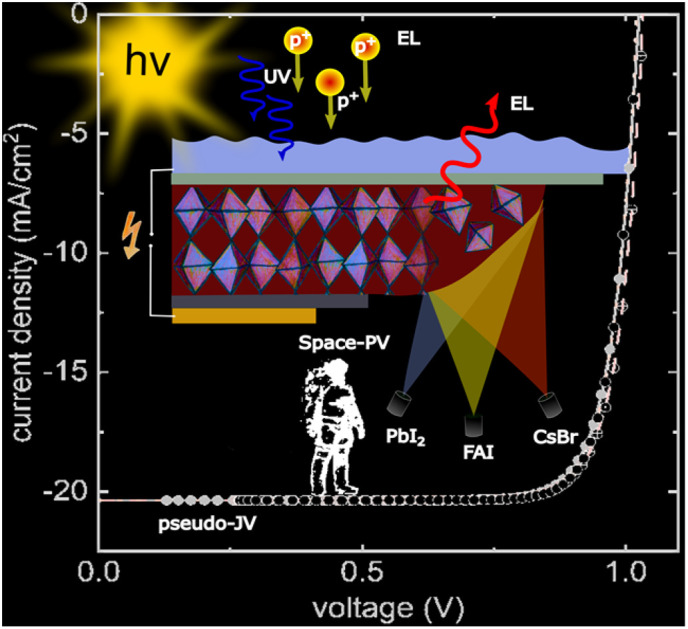

## 1 Introduction

Considering Earth's large gravity well, large-scale space infrastructures have to be assembled or built-in space or on the Moon. A crucial component of any moon base, space station, or spacecraft is its energy supply, and therefore, the future fabrication of solar cells in space is of high interest. The manufacturing of silicon solar cells in space or on the Moon has been proposed and discussed extensively.^1,2^ In fact, regolith found on the Moon contains plenty of silicon, and therefore, *in situ* resource utilization also seems possible.^1^ However, both the refinement of regolith to Si with the necessary purity, as well as the fabrication of silicon solar cells, requires high temperatures >1400 °C and is very energy-demanding and complex. Even when fabricated on earth, silicon solar cells have a non-negligible energy payback time (EPBT), around 1–2.5 years, depending on installment location, fabrication *etc.*^3–5^

In this work, we propose and test a halide-perovskite-based photovoltaic technology that could be easily fabricated in space by physical vapor deposition, making use of the available vacuum present. Using evaporation, a variety of surfaces, be it the outside of a space station or the moon surface itself, could be turned into PV modules. With processing temperatures <150 °C, halide perovskite-based solar cells offer much lower energy payback times of ∼0.2–1.5 years depending on the architecture, fabrication, and installment.^3,5^ Typical halide perovskites further tolerate a relatively high number of impurities (‰ to %)^6^ without losses in performance (compared to <ppb in Si),^7^ lowering the requirements for deposition and precursor quality. Even despite that, solution-processed halide perovskite-based solar cells have reached power conversion efficiencies >25%,^8^ rivaling those of traditional PV materials.^8^ Typical halide-based perovskites possess large absorption coefficient and thus ultra-thin absorber layers ∼500 nm thick are sufficient for efficient photovoltaics. Just 1 kg of perovskite precursors brought into space could be used to coat around 400 m^2^, though we note that this estimate excludes additional contact and interlayers and is only meant to exemplify the benefit of in-space manufacturing of ultrathin-large area space PV solutions. More detailed considerations have recently been discussed by McMillon-Brown *et al.*^9^

Many solution-based perovskites have been investigated recently regarding their radiation tolerance, a crucial requirement for any space application.^10–13^ In orbit, especially outside the earth's magnetosphere, on the Moon, and beyond, harsh particle irradiation is an omnipresent threat to any astronaut, satellite, electronics, and space PV module. Recently we have investigated the radiation hardness of perovskite-based tandem PV and shown that both perovskite/CIGS^14^ and perovskite/perovskite^15^ tandems are attractive and exceed the radiation hardness of traditional space PV systems.

Herein, we focus on the proton radiation hardness of a fully evaporated perovskite composition to investigate and show that evaporated perovskites are a radiation-tolerant option for in-space manufactured PV systems. Interestingly, fully evaporated perovskites still do not reach efficiency and lifetime records of their solution-processed counterparts, see *e.g.* Fig. S1,† possibly due to imperfect stoichiometries as a result of poor evaporation control, varying sticking coefficients, dissociation of organic cations during evaporation or low crystallinities/poor growth, parasitic phases, residual strain, or even absence of solvent residues.^16,17^ These issues potentially could also indicate a lower radiation tolerance. Our experiments nevertheless demonstrate that co-evaporated perovskite layers are resistant to the harsh radiation environment in space on par with their solution-processed counterparts.^11,18–24^ Notably, we extract this information from pseudo-light *JV* curves extracted from electroluminescence (EL) and Suns-*J*_SC_–*V*_OC_ measurements that are essentially unaffected by the parasitic darkening of typical glass/ITO substrates under irradiation. Our methodology thus does not require special radiation-resistant glasses (those rely, for example, on Ce-doping but are far less common and significantly more expensive) and thus might help to speed up research on novel radiation-tolerant material systems. Moreover, the evaporated perovskite absorbers are an ideal system to investigate the impact of radiation-induced defects – after all, they are more uniform than their solution-processed counterparts (facilitating high-spatial-resolution microscopies) and remain free from solvent residues (that could play a role in the degradation and self-healing mechanisms). Lastly, we would like to emphasize that many further tests such as atomic oxygen exposure, thermal vacuum, temperature cycles *etc.* according to IEC/ISO standards are necessary to validate evaporated perovskite solar cells for future space applications.

## 2 Results

**Fig. 1:**
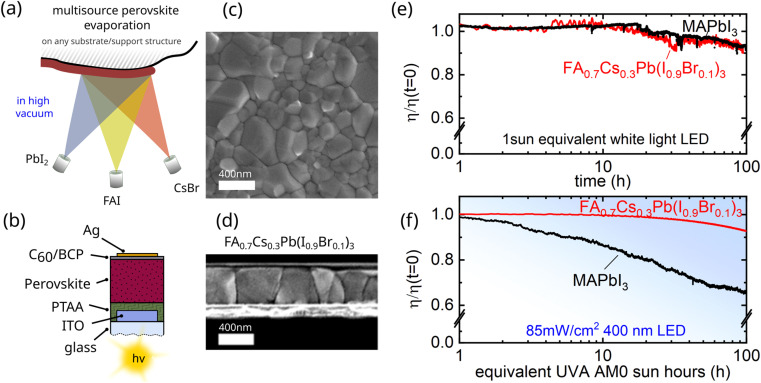
Multisource vacuum deposition of halide perovskite absorbers (a) illustration of multisource vacuum deposition of high-quality perovskite absorbers comprising FA_0.7_Cs_0.3_Pb(I_0.9_Br_0.1_)_3_ from PbI_2_, FAI, and CsBr. (b) Device structure and (d) cross-sectional micrograph of the here used device structure comprising glass/ITO/PTAA/FA_0.7_Cs_0.3_Pb(I_0.9_Br_0.1_)_3_/C_60_/BCP/Ag. (c) Depicts a top-view micrograph of the evaporated perovskite absorber on glass/ITO/PTAA. (e) Long-term MPP tracking results of evaporated FA_0.7_Cs_0.3_Pb(I_0.9_Br_0.1_)_3_ and spin-coated MAPbI_3_ based solar cells under white light LED 1 sun equivalent illumination or 400 nm deep blue/UV LED illumination (f).

To demonstrate the suitability of fully evaporated perovskite semiconductors/absorbers for space applications, we deposited by thermal evaporation an FA_0.7_Cs_0.3_Pb(I_0.9_Br_0.1_)_3_ based perovskite absorber layer.^16^ The co-evaporation of PbI_2_, formamidinimium-iodide (FAI), and CsBr hereby allows conformal coating with precise control over the thickness, see Fig. 1a–d. Devices fabricated in a p-i-n configuration comprising glass/ITO/PTAA/FA_0.7_Cs_0.3_Pb(I_0.9_Br_0.1_)_3_/C_60_/BCP/Ag with PTAA being poly[bis(4-phenyl)(2,4,6-trimethylphenyl)amine] and BCP being bathocuproine reach champion efficiencies of ∼18%, as we reported recently,^16^ and average efficiencies ∼15–16% are still among the highest for reproducible fully evaporated methylammonium free perovskites.^25^ Using a methylammonium-free perovskite absorber further minimizes UV degradation effects otherwise occurring in typical perovskite absorbers.^26–28^ To illustrate this, we compare the degradation of the archetypical (spin-coated) MAPbI_3_ to the here fabricated FA_0.7_Cs_0.3_Pb(I_0.9_Br_0.1_)_3_ based devices under white light *vs.* intense UVA light in Fig. 1e and f, respectively. It can clearly be seen that using FACs instead of MA eliminates a UV degradation mechanism, which is crucial for any application in space, as the extra-terrestrial solar spectrum (AM0) contains a considerably larger UV proportion. Note that under white light, both compositions degrade similarly, which we suspect is due to a PTAA-related degradation channel that has been revealed recently and which can be mitigated using self-assembled monolayers (SAM) to fabricate long-term stable devices.^29,30^ We further fabricated MA-free spin-coated perovskites that corroborate that the enhanced UV stability stems form the absence of MA and not from the absence of solvent residues, see Fig. S3 and S4.†

**Fig. 2:**
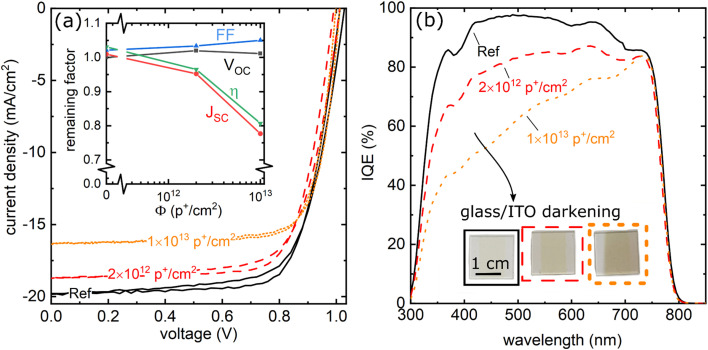
Degradation of evaporated perovskite device performance under 68 MeV proton irradiation (a and b) *JV* characteristics, evolution of the remaining factor (*i.e.* performance metric ratio before and after degradation) of *V*_OC_, *J*_SC_, FF and *η* (a) and internal quantum efficiency (b) after 2 × 10^12^ p^+^ cm^−2^ and 1 × 10^13^ p^+^ cm^−2^ compared to a non-irradiated reference device. The photograph in (b) further shows the darkening of the used glass substrates being responsible for the IQE losses at low wavelengths <700 nm. While *J*_SC_ and reduced according to the reduced glass transmission, remarkably no degradation in *V*_OC_ and FF is observed.

We then tested the radiation tolerance of the evaporated FA_0.7_Cs_0.3_Pb(I_0.9_Br_0.1_)_3_ based solar cells under high energetic 68 MeV proton radiation that is not only capable of inducing ionizing as well as displacement damage throughout the device but also allows to irradiate though the encapsulation glass^12,15^ thereby minimizing parasitic degradation effects from O_2_ or H_2_O. As seen in Fig. 2a, the efficiency of the tested solar cells degraded to ∼0.8 of their initial efficiency due to degradation of the *J*_SC_. At the same time, the FF and *V*_OC_ remain unaffected. Within the investigated timescale, the degradation in *J*_SC_ is solely due to the creation of color centers within the glass substrate, reducing the EQE below 700 nm, as seen in Fig. 2b. Parasitic glass coloring is a well-known effect that reduces the transmission of regular glass between 300 nm to 700 nm (see also Fig. S5a†). This, in turn, can easily dominate the degradation of otherwise radiation-tolerant PV technologies. To solve this problem, radiation-hard quartz or Ce-doped glasses can be used; both are, however, expensive, difficult to source, and not standardly used for solar cell research. Interestingly, we found that several flexible substrates, such as PET/ITO (Fig. S5b and S6†) are unaffected and do not discolor upon proton irradiation. While the development of flexible devices is beyond the scope of this paper, they present an attractive low-cost option for future use.

**Fig. 3:**
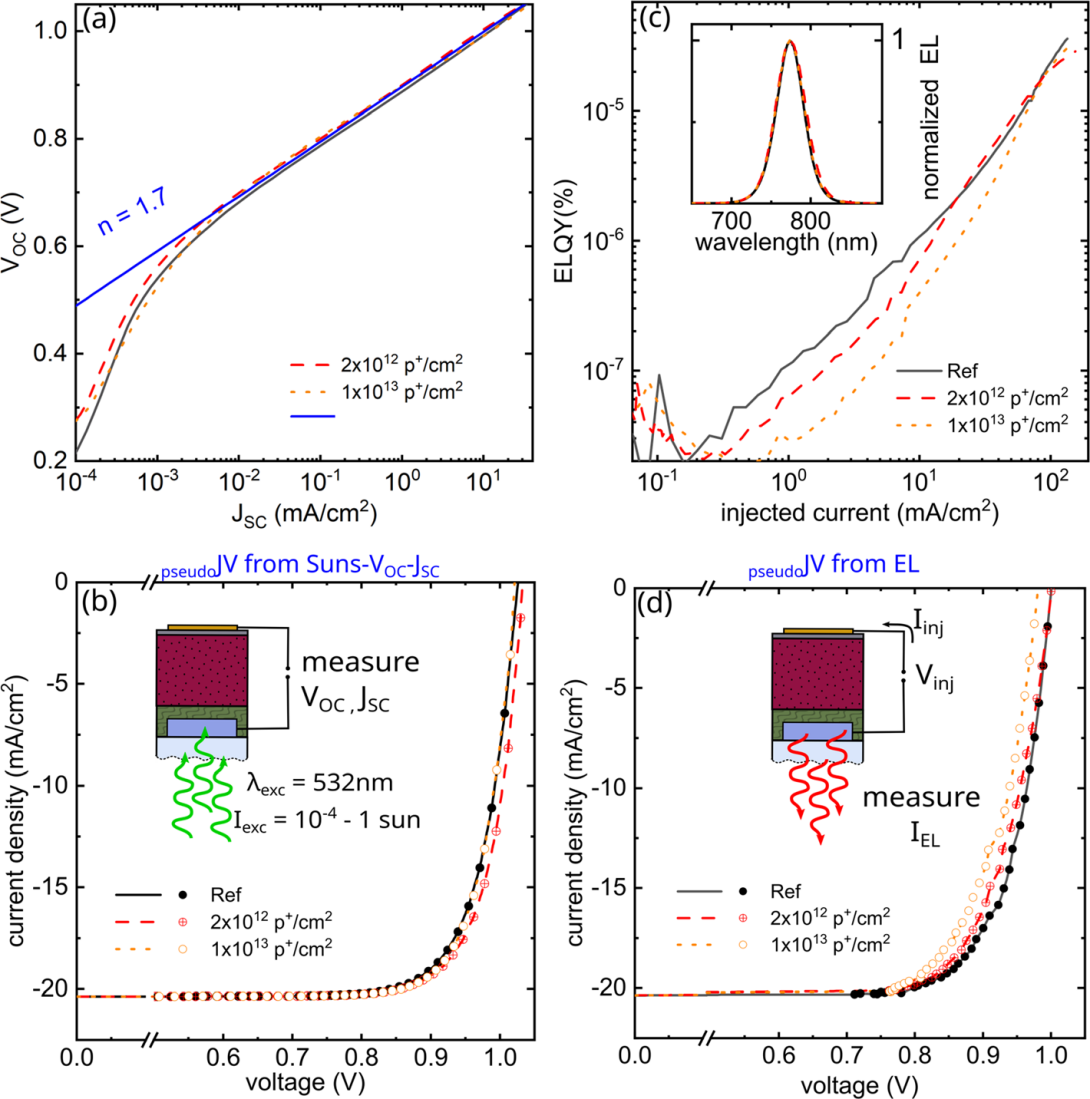
Radiation hardness of vacuum-deposited FA_0.7_Cs_0.3_Pb(I_0.9_Br_0.1_)_3_ (a) *V*_OC_ as a function of *J*_SC_ (Suns-*V*_OC_–*J*_SC_) under *λ* = 532 nm laser illumination with varying intensity corresponding to 10^−4^ to 1 sun for reference and proton irradiated devices. Irradiated and non-irradiated measurements collapse on a single trend suggesting negligible radiation-induced damage. (b) Pseudo *JV* characteristics derived from Suns-*V*_OC_–*J*_SC_. (c) Electroluminescence quantum efficiency EQE_EL_ as a function of injection current for reference and proton irradiated devices. The inset depicts the normalized EL spectrum. (d) Pseudo *JV* characteristics derived from EL.

In the following, we record and examine pseudo-light-*JV* characteristics from (i) Suns-*J*_SC_–*V*_OC_ and (ii) electroluminescence (EL) and that allow us to assess the radiation-induced degradation without being affected by the parasitic glass coloring described above. We start with Suns-*J*_SC_–*V*_OC_, where we record the *V*_OC_ and *J*_SC_ for a variety of different light intensities between 10^−4^ and 1 sun. By plotting the *V*_OC_*vs. J*_SC_, we can then compare the *V*_OC_ at identical generation currents. We note that depending on the glass coloring, different light intensities were needed to generate the same *J*_SC_. As seen in Fig. 3A, measurements on reference and irradiated devices collapse on an identical trend, following an ideality factor of ∼1.7 with some shunt-dominated behavior at low currents. Flipping the *x*-and-*y* axis allows us to plot the same data now as a pseudo-dark *JV* curve, and by subtracting the light-induced generation current of ∼20 mA cm^−2^, we generate a pseudo-light *JV* curve. As shown in Fig. 3b, those again collapse on an identical trend, with identical FF and *V*_OC_, indicating the optoelectronic properties of the perovskite remain high and unaffected by the high energetic proton irradiation.

We then continue our analysis with the EL measurements, which are unaffected by the glass colouring, as the emission wavelength at 775 nm falls outside the typical glass colour centers created upon proton irradiation (see *e.g.* Fig. S5 and S6†). As shown in Fig. 3c, we, therefore, observe identical EL spectra before and after irradiation. Interestingly, however we observe a slight decrease of the electroluminescence quantum yields (ELQY) at low injection currents that could point to some radiation damage of the active layer stack. At high injection currents (*J*_inj_) of ∼20 mA cm^−2^, however, the ELQY reach again similar values of 2.2 × 10^−4^% (reference), 2.2 × 10^−4^% (2 × 10^12^ p^+^ cm^−2^), and 1.1 × 10^−4^% (1 × 10^13^ p^+^ cm^−2^). We then can calculate the quasi-Fermi level splitting (QFLS) from the measured ELQY for each *J*_inj_ using eqn (1)1
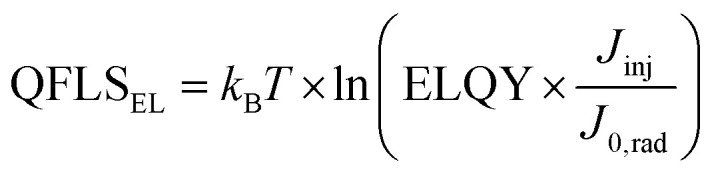
with *J*_0,rad_ being the radiative dark recombination current. For the reference and irradiated devices at the two doses, this yields QFLS values of 1.00, 1.00, and 0.98 eV, respectively, indicating some minor damage from proton irradiation at the highest dose measured.

Plotting the internal voltage (iV_EL_ = QFLS_EL_/*e*) on the *x*-axis and the *J*_inj_ current minus *J*_SC_ (*J* = *J*_inj_ − *J*_SC_) on the *y*-axis again allows us to derive pseudo-*JV* curves, as plotted in Fig. 3d. Comparing reference and proton-irradiated devices shows a slight reduction of *V*_OC_, pseudo-FF (pFF), resulting in somewhat reduced pseudo-efficiencies of 0.98 and 0.96 of the non-irradiated devices after doses of 2 × 10^12^ p^+^ cm^−2^ and 1 × 10^13^ p^+^ cm^−2^. We summarize all remaining factors in Table 1 and note that these performance losses are small compared to conventional III–V on Ge triple junctions solar cells (remaining efficiency <0.8 at 1 × 10^13^ p^+^ cm^−2^, 68 MeV) that we recently irradiated under identical conditions.^15^ For completeness, we note that both pseudo-*JV* characteristics are free of parasitic transport losses, however, we can exclude these changes upon proton irradiation as standard *JV* measurements under 100 mW cm^−2^ discussed before even reveal a slight increase in FF.

**Table 1:** Performance metric ratios before and after degradation of vacuum deposited FA_0.7_Cs_0.3_Pb(I_0.9_Br_0.1_)_3_ perovskites. Values were determined from *JV*, Suns-*V*_OC_–*J*_SC_, or EL as indicated

	Remaining factors	
	2 × 10^12^ p^+^ cm^−2^	1 × 10^13^ p^+^ cm^−2^
**From *JV* under 100 mW cm^−^** ^**2**^		
*V*_OC_	1.02	1.01
*J*_SC_	0.95	0.78
FF	1.01	1.03
PCE	0.94	0.79

**From *J***_**SC**_ **− *V***_**OC**_		
iV_OC_	1.01	0.995
pFF	1.01	1.02
pPCE	1.01	1.01

**From EL**		
iV_OC_	1	0.98
pFF	0.98	0.98
pPCE	0.98	0.96

**Fig. 4:**
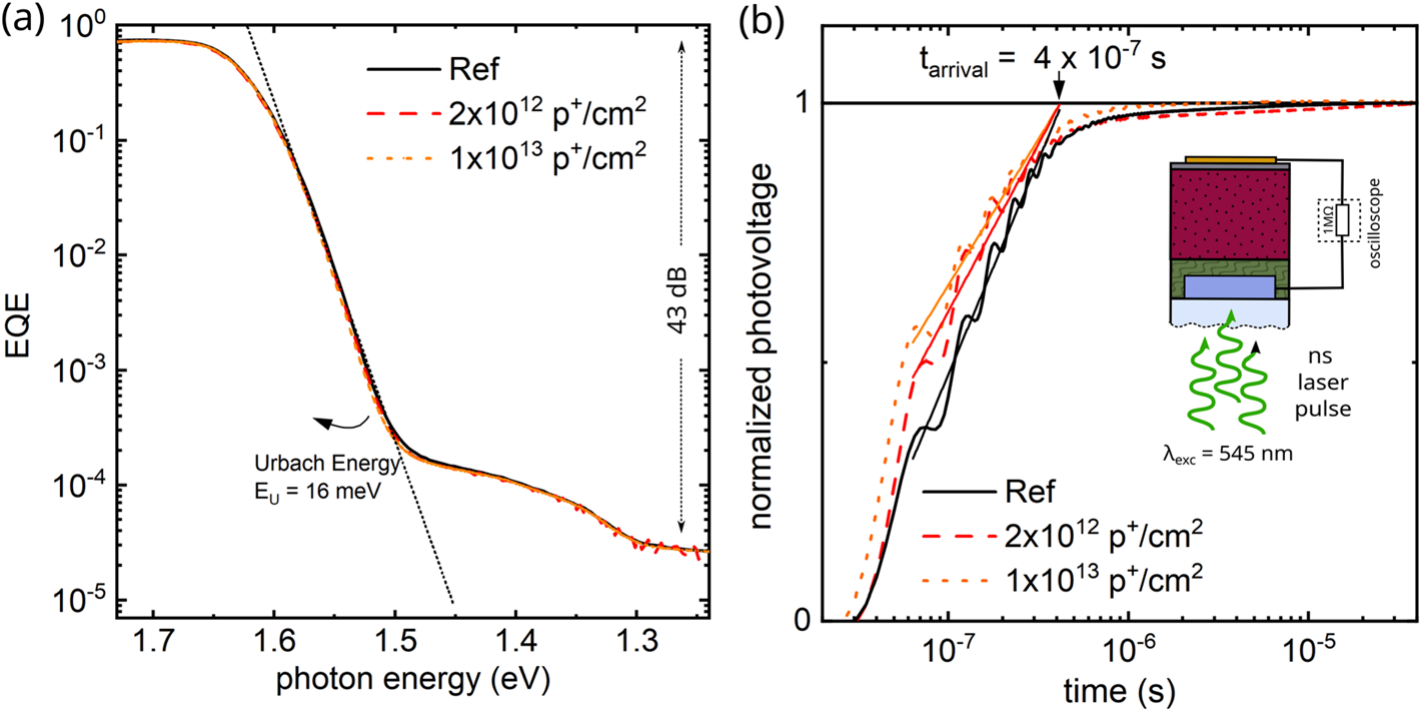
Defects and charge extraction after proton irradiation (a) external quantum efficiency *vs.* incident photon energy of reference and proton irradiated devices. The signal-to-noise ratio was above 46 dB. The black dotted line represents the exponential drop below the band gap as expected from an Urbach energy of 16 meV. The disorder is therefore unaffected by proton irradiation. (b) Normalized transient photovoltage of reference and proton irradiated devices measured upon excitation with an ns laser pulse. The signal is measured across a load resistance of 1 MΩ, and thus the signal saturates once the photo-excited charge carriers reach the respective electrodes. The arrival time, however, does not change upon proton irradiation.

Since the *V*_OC_, FF, and ideality factor of our evaporated perovskite solar cells are apparently barely impacted by high energetic proton irradiation, we began an in-depth analysis to identify potential radiation-induced damage. Firstly, we looked at the disorder of the perovskite absorber using sensitive external quantum efficiency measurements shown in Fig. 4a. When analyzing the Urbach energy (of around 16 meV), however, we did not find any change upon irradiation, again suggesting that the disorder within the perovskite absorber remains unaffected by proton irradiation. Then, we measured resistance dependent photovoltage (RPV) transients to analyze if charge extraction is changed upon proton irradiation.^31,32^ This could be caused by damaged interfaces, interlayers, or the respective electron and hole transport layers and has been observed in perovskite/spiro-OMeTAD based devices^19^ as well as all-perovskite tandem solar cells utilizing a LiF/C_60_/AZO/ITO/PEDOT:PSS recombination layer.^15^ For RPV, we use a short (ns-long) laser pulse to excite the samples and then record the photovoltage build-up *via* an oscilloscope and a large 1 MΩ resistor. Consistent with the above p*JV* results, we did not observe significant differences in the arrival time of the charge carriers at the respective electrodes and therefore concluded that also the used charge transport layers are relatively unaffected and not damaged.

**Fig. 5:**
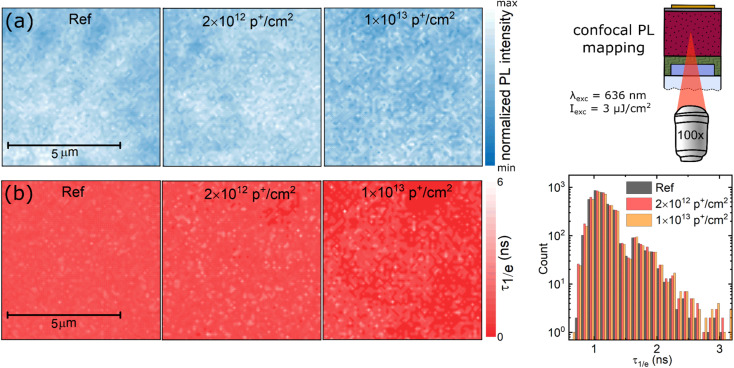
High-spatial resolution photoluminescence imaging of proton irradiated evaporated perovskite solar cells (a) confocal photoluminescence intensity map of the perovskite absorber. As indicated, excitation was performed through a 100× long working distance objective using a 636 nm excitation at 3 μJ cm^−2^. For comparison, the PL intensity was normalized for each image. This was necessary to account for the reduced glass transmission due to color centers created upon proton irradiation (b) PL lifetime maps and histogram of the 1/*e* lifetime (*τ*_1/*e*_), indicating no significant change upon proton irradiation. Corresponding PL decays, as well as additional decays measured under lower excitation fluence, are shown in the ESI, Fig. S8 and S9.†

Lastly, we searched for local defect clusters, which can be seen around the ion track of high energetic particles^15,33,34^ using high spatial resolution confocal photoluminescence imaging. Recorded PL and PL lifetime maps reveal typical heterogeneities of poly-crystalline perovskite absorbers already in non-irradiated reference devices; see Fig. 5a and b. When comparing PL maps recorded on irradiated specimens to control devices, we observe no increase in apparent defect clusters, which is further corroborated by the unchanged PL lifetimes obtained. Note that we do observe local defect clusters after identical irradiation conditions in III–V based semiconductor materials that are routinely used for space applications.^15^ The absence of radiation induced damage suggest that damage from high-energetic protons is strongly suppressed in halide perovskites. Currently, we hypothesize that the softer lattice of halide perovskites and low-migration barriers of ions, vacancies, and interstitials^35^ allow swift relaxation (or healing) of local lattice defects.

## 3 Conclusion

In summary, we have examined the radiation tolerance under high energetic proton irradiation of novel solar cells made using evaporated perovskite absorbers. We find high remaining factors (∼1) of *V*_OC_ and FF using standard *JV* characterization. At the same time, the *J*_SC_ reduces upon apparent transmission losses from radiation-induced color centers within the glass substrates, a common problem that can be avoided using expensive radiation-tolerant substrates. We then establish and test a variety of techniques to access and quantify potential radiation-induced damage of the perovskite itself. For this purpose, we derive pseudo-*JV* characteristics from Suns-*J*_SC_–*V*_OC_ and injection-dependent EL measurements. These quantities allow us to assess performance losses even in spite of the glass coloring. Our characterizations reveal high remaining efficiencies (>0.96 at 1 × 10^13^ p^+^ cm^−2^) beyond today's space material systems (<0.8). Even using more in-depth characterization techniques, we cannot observe any signature of radiation-induced damage. Together our findings show the potential of evaporated perovskite solar cells potentially fabricated in space to power spacecraft or future bases on the Moon, yet further tests according to IEC/ISO standards are necessary to validate this technology for future space application.

## Author contributions

F. L. initiated the research and planned the experiments with input from M. S. and S. D. S. Y.-H. C. fabricated all perovskite solar cells. Y.-H. C. and F. L. performed the photovoltaic characterizations. F. L. recorded Suns-*V*_OC_–*J*_SC_, EL, EQE, UV-VIS, and RPV measurements. S. Ö. fabricated and tested solution processed MA-free perovskites. F. L. further recorded PL lifetime maps and K. F. helped with lifetime calculations. F. L. analyzed all data and took the lead in drafting the manuscript. F. L. and S. D. S. wrote the paper with input from other authors. All authors contributed to the discussion of the results.

## Conflicts of interest

S. D. S. is co-founders of Swift Solar, Inc., a company commercializing high-power, lightweight perovskite solar panels.

## Supplementary Material

RA-013-D3RA03846G-s001
