# Supplementary material for: Methylammonium-free co-evaporated perovskite absorbers with high radiation and UV tolerance: an option for in-space manufacturing of space-PV?
Source: RSC Adv. 2023 Jul 12;13(31):21138–45. doi: 10.1039/d3ra03846g (PMC10337721; doi:10.1039/d3ra03846g)
Supplement: RA-013-D3RA03846G-s001 [file RA-013-D3RA03846G-s001.pdf]

Electronic Supplementary Material (ESI) for RSC Advances.
This journal is © The Royal Society of Chemistry 2023

Supplementary Information

Methylammonium-free co-evaporated perovskite absorbers with high radiation and UV tolerance: An option for in-space manufacturing of space-PV?

Felix Lang[1,2], Yu-Hsien Chiang[1], Kyle Frohna[1], Sercan Özen[2], H. C. Neitzert[3], A. Denker[4,5], Martin Stolterfoht[2], Samuel D. Stranks[1,6*]*

[1] Cavendish Laboratory, Department of Physics, University of Cambridge, JJ Thomson Avenue, CB3 0HE, Cambridge, UK

[2] Institute of Physics and Astronomy University of Potsdam Karl-Liebknecht-Str. 24–25, Potsdam-Golm D-14476, Germany

[3] Department of Industrial Engineering (DIIn), Salerno University, Fisciano (SA), Italy

[4] Helmholtz-Zentrum Berlin für Materialien und Energie GmbH, Protonen für die Therapie, Hahn-Meitner Platz 1, 14109 Berlin, Germany

[5] Beuth Hochschule für Technik Berlin, Fachbereich II – Mathematik – Physik – Chemie, Luxemburgerstr. 10, D-13353 Berlin, Germany

[6] Department of Chemical Engineering & Biotechnology, University of Cambridge, Philippa Fawcett Drive, CB3 0AS, Cambridge, UK

* lang1@uni-potsdam.de, sds65@cam.ac.uk

Keywords: in-space manufacturing, perovskite, solar cell, radiation hardness, space photovoltaics, stability, radiation-induced defects, degradation, proton-irradiation

Supplementary Information

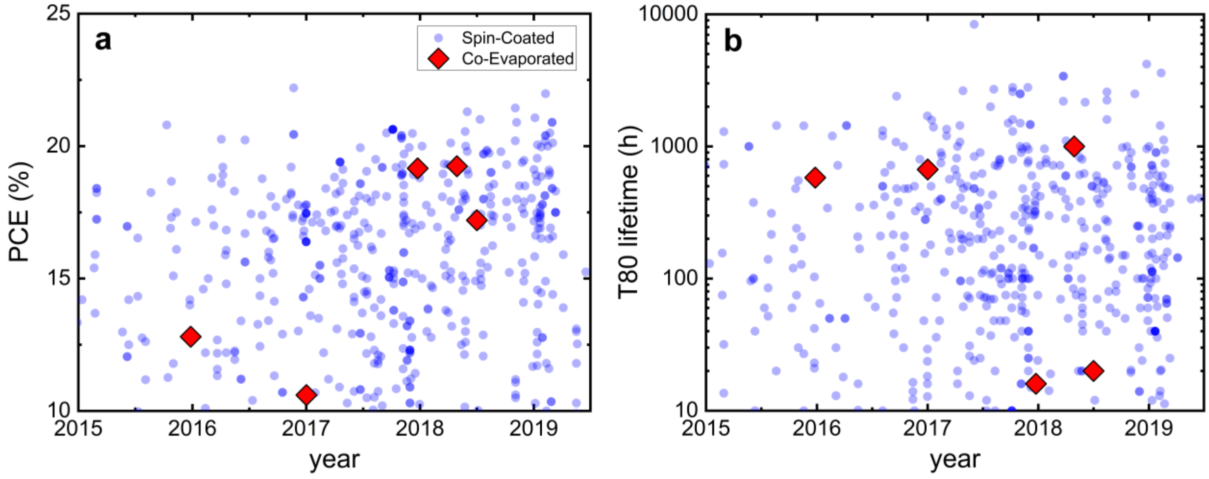

Fig S1: Power conversion efficiency (a) and T80 lifetime of spin-Coated vs Co-Evaporated Perovskite Solar Cells. Data extracted from the Perovskite Database. [1]

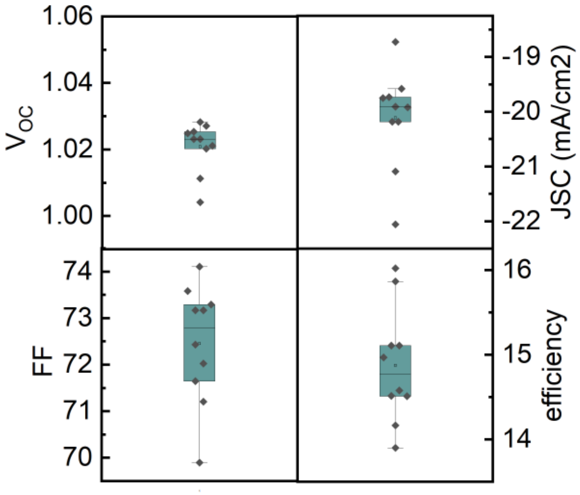

Fig. S2. Summary of relevant PV parameters of the investigated devices non-irradiated vacuum processed $FA_{0.7}Cs_{0.3}Pb(I_{0.9}Br_{0.1})_3$ processed perovskite solar cell.

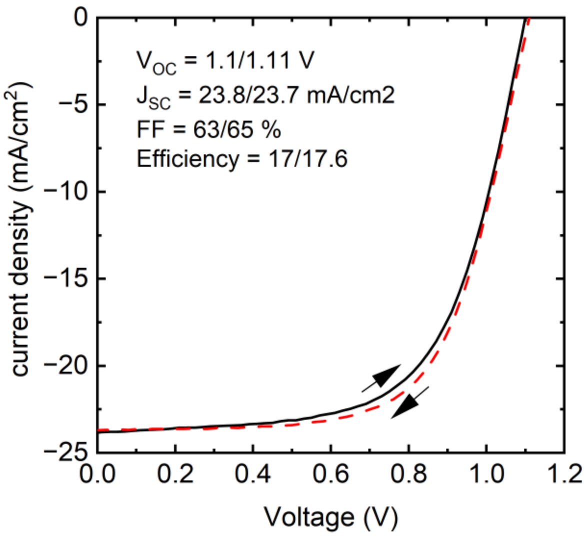

Fig S3: JV Characteristics of solution processed MA-Free Perovskite

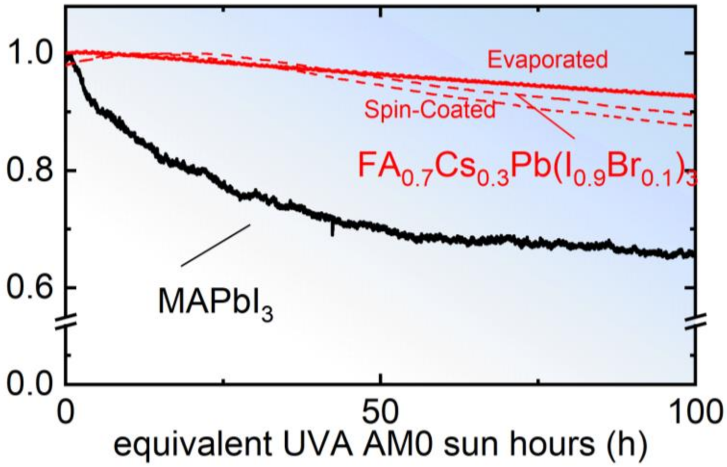

Fig S4: UV-Degradation of spin-coated and evaporated MA-Free Perovskites vs MAPbI3 (spin-coated)

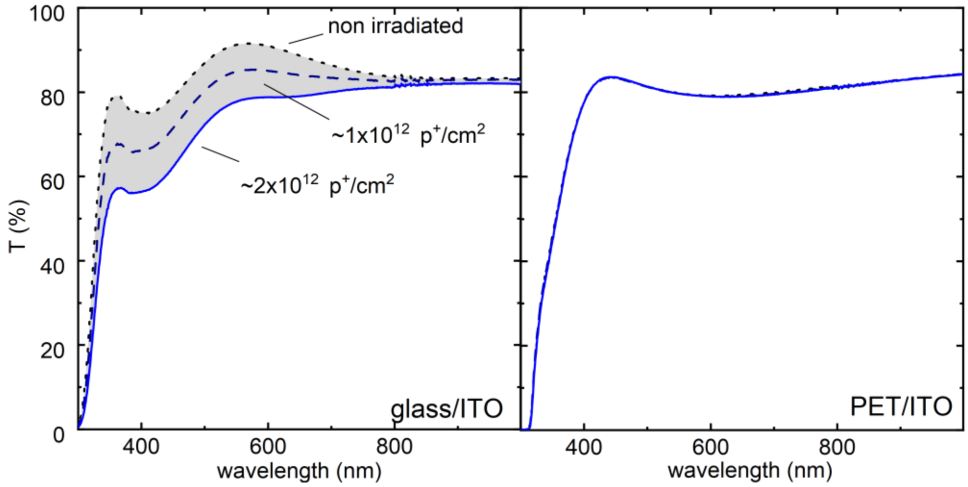

Fig. S5. Transmission of a typical glass/ITO substrate vs a PET/ITO foil before and after high energetic proton irradiation.

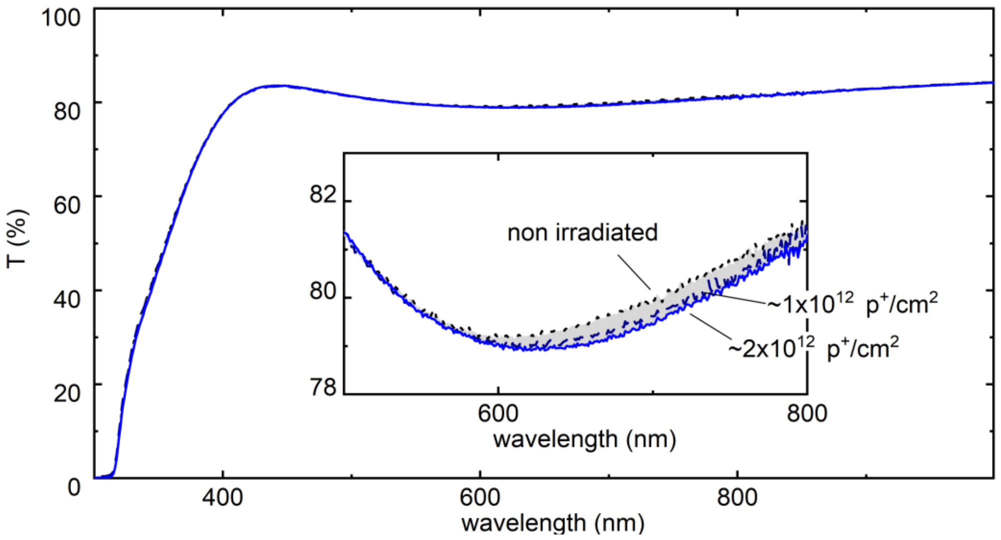

Fig. S6: Transmission of PET/ITO before and after proton irradiation.

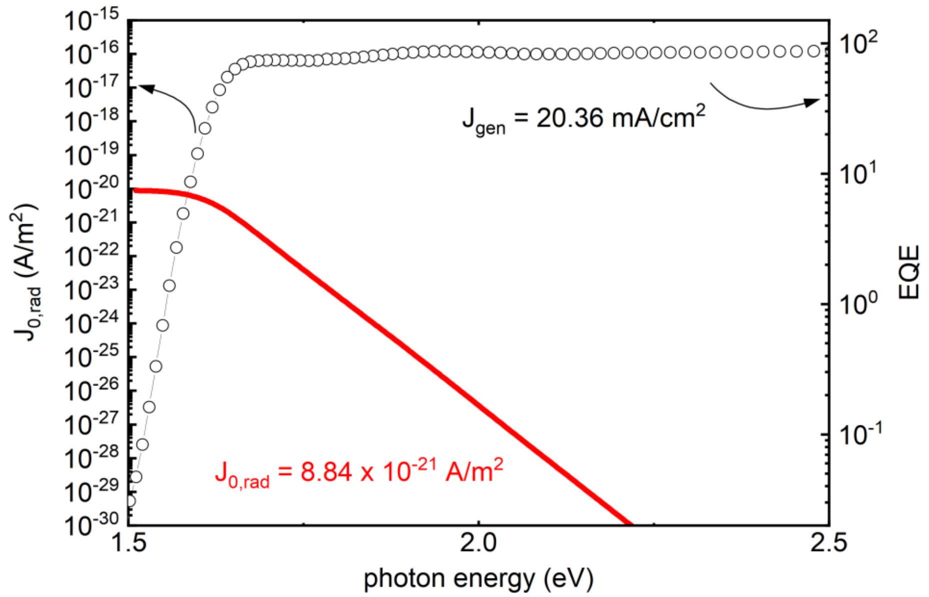

Fig. S7. $J_{0,gen}$ **and** J_{gen} **of non-irradiated vacuum processed** $FA_{0.7}Cs_{0.3}Pb(I_{0.9}Br_{0.1})_3$ **processed perovskite solar cell.**

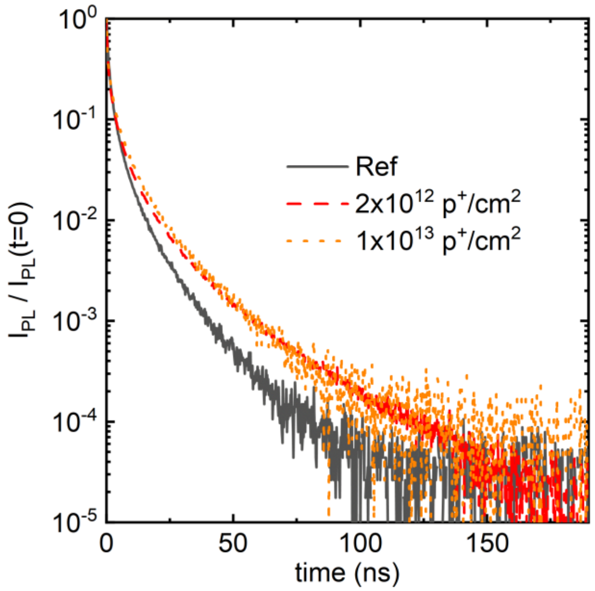

Fig. S8. Transient photoluminescence decays of reference and proton irradiated devices. Excitation was performed using a pulsed 636 nm laser at an intensity of 3 µJ/cm^2 though a 100x objective.

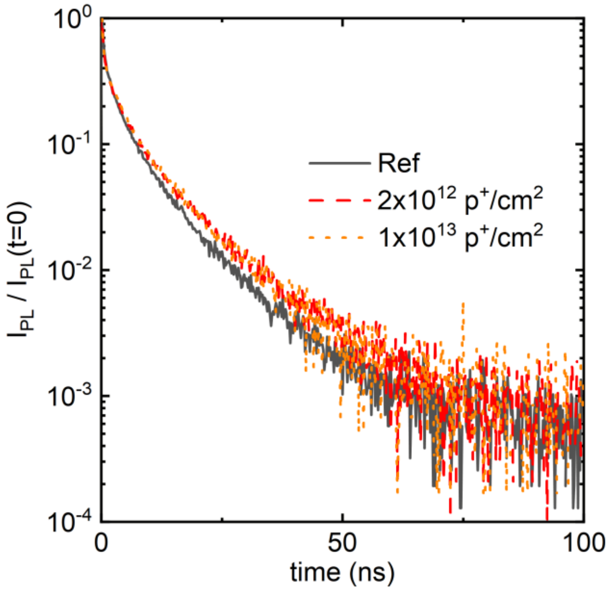

Fig. S9. Transient photoluminescence decays of reference and proton irradiated devices. Excitation was performed using a pulsed 636 nm laser at an intensity of 1 nJ/cm^2 though a 20x objective.

Experimental Procedures

Vacuum Deposition of Perovskite Solar Cells*:* --- To fabricate the perovskite solar cells we cleaned ITO (150 nm) coated glass substrates using soap, deionized water, acetone and isopropanol and UV-Ozone and then spin-coated a thin layer of PTAA (Sigma, 2mg/ml in toluene at 5000rpm for 30s) as bottom hole transport layer inside a nitrogen filled glovebox. Without exposure to oxygen the samples were then transferred to a CreaPhys PEROvap system and immediately evacuated to a base pressure of 1-3x10-6 mbar for subsequent perovskite deposition. Note that the PEROvap system employs a specifically designed cooling system that maintains the evaporator walls, source shutters and shields at -20ºC throughout the entire process to minimize re-evaporation of the pre-cursors and cross-contamination between sources, ensuring fine control over the evaporation. For mixed cation lead mixed halide deposition, we then evaporated FAI (GreatCell Solar), Cesium bromide (Sigma) and PbI_2 (TCI or Lumtec) to yield the final composition of $FA_{0.7}Cs_{0.3}Pb(I_{0.9}Br_{0.1})_3$ with 10% PbI_2 excess. Further details on perovskite evaporation are given in ref[2]. After the perovskite deposition, films were annealed at 135ºC for 90 min. After cooling down to room temperature, we spin-coated PCBM (Merck) (20 mg/ml in anhydrous Chlorobenzene, sigma) onto the perovskite films at 1200 rpm for 30 seconds and left to dry at room temperature in the glovebox for 20 minutes. Lastly, we spin coated Bathocuproine (BCP; Alfa Aesar), dynamically at 2000 rpm for 30 seconds and evaporated 100 nm of Ag at 1 Å/s.

Spin-Coating of Perovskite Solar Cells: In order to create spin-coated perovskite solar cells, we began by cleaning patterned ITO substrates (2.5×2.5 cm^2, 15 Ω/sq, Psiotec, UK) through a series of sonication steps in acetone, Hellmanex solution in deionized water (DI), DI water, isopropanol (IPA), and acetone. The sonication durations were established as 2 minutes for Hellmanex solution and deionized water, while 10 minutes were allotted for acetone and isopropanol (IPA). The substrates were then dried under N2 flow, treated with oxygen plasma for 30 minutes, and immediately transferred into the N2-filled glovebox. For the HTL, we dropped 60 µL of 1.75 mg/mL PTAA (Poly-[bis-(4-phenyl)-(2,4,6-trimethylphenyl)-amin], Sigma-Aldrich) solution in toluene onto a sample and spin-coated for 30 seconds at 6000 rpm with 2000 rpm/s acceleration. This was followed by annealing on a hotplate at 100°C for 10 minutes. After cooling down to room temperature, 60 µL of 0.5 mg/mL PFN-Br (Poly(9,9-bis(3'-(N,N-dimethyl)-N-ethylammoinium-propyl-2,7-fluorene)-alt-2,7-(9,9-dioctylfluorene))dibromide, 1-Metarial) solution in methanol was dynamically spin-coated on top of the PTAA layer at 4000 rpm for 30 seconds without any annealing process followed. To create the perovskite film on the HTL-coated substrates, we deposited 120 µL of perovskite solution onto the substrate and statically spin-coated at 1000 rpm for 10 seconds and at 4000 rpm for 30 seconds. 12 seconds before the spin-coating ended, we continuously dripped 250 µL chlorobenzene onto the center of the film. Afterward, heating at 110°C was applied for 10 minutes. To achieve the resulting $Cs_{0.05}(MA_{0.02}FA_{0.98})_{0.95}Pb(I_{0.98}Br_{0.02})_3$ stoichiometry, we added MABr, MACl, CsI, FAI, and PbI2 into the 1:5 DMSO/DMF mixture and stirred for 4 hours at 55°C in N2 filled glovebox. We achieved an MA-free perovskite solution by replacing the MABr with FABr. All other ingredients remained constant and were also solved in the DMSO/DMF mixture in the glovebox. Lastly, in order to form an ETL, we deposited C60 (30 nm) and then 2,9-Dimethyl-4,7-diphenyl-1,10-phenanthroline BCP (8 nm) on the samples. The top

contact was created by evaporating copper on top and 100nm copper deposited onto the ETL. All evaporation processes were performed under a vacuum (p = 10-7 mbar). By the fabricated device structure, the area of each pixel is defined by the overlapping area of the ITO electrodes and the copper (6 mm^2).

Proton Irradiation*:* Proton irradiation experiments were performed at the proton accelerator complex of the Helmholtz-Zentrum Berlin[3,4]. The proton energy amounted to 68 ± 1 MeV, and the irradiation area was about 3.0 cm^2, defined by aperture masks. Thin scattering foils ensured a homogenous irradiation. The beam intensity was monitored online, utilizing a transmission ionization chamber. After irradiation, all devices were transferred into a lead cabinet. After one week, the remaining activity of the generated short-living isotopes was measured to confirm that the activity dropped to a safe level of less than 10^3 Bq. In total, this amounted to about ten days of storage before post-irradiation characterizations could be performed. A non-irradiated reference device was used to track (barely existent) changes due to prolonged sample storage.

Current-Voltage-Characteristics Current-voltage scans were recorded in forward and reverse directions with a voltage sweep of 85 mV/s under illumination using a Wavelabs Sinus 70 AAA LED sun simulator. Simulated AM1.5G and AM0 spectra are shown in Figure S9. We adjusted the intensity to 100 and 135 mW/cm^2 respectively by measuring the short-circuit current of a calibrated silicon solar cell (Fraunhofer ISE). In addition to forward and reverse measurements, we tracked the maximum power point (MPP) using homemade feedback software over 3 minutes. In all cases, the temperature amounted to 25°C. In the case of the perovskite 2J tandem solar cells, we employed shadow masks (A = 0.058 cm^2) to avoid underestimation of the active area and overestimation of the J_{SC}.

Suns-V_{OC}-J_{SC} Measurements: Steady-state intensity dependent $V_{OC} - J_{SC}$ measurements were obtained with a 520 nm continuous wave laser from Insaneware providing a power of up to 1 W. A continuously variable neutral density filter wheel from ThorLabs then was used to attenuate the laser power (up to OD 6). The light intensity was thereby simultaneously measured with a silicon photodetector and a Keithley 485 to improve the accuracy of the measurement. The measurement was performed by measuring alternating the V_{OC} at a given intensity and then the J_{SC} before the filterwheel rotated to the next position using a custom-built Labview code. To check whether the V_{OC} reached steady-state conditions, the temporal evolution of the open-circuit voltage and the J_{SC} was recorded.

Electroluminescence (EL) Measurements: Absolute EL was measured with a calibrated Si photodetector from Newport using a Keithley 485 pico Ampere meter. The detector (with an active area of ~2 cm^2) was placed directly in front of the device (< 0.5 cm) and the total photon flux was

evaluated considering the emission spectrum of the solar cell and the external quantum efficiency of the detector (around 86 % in the relevant spectral regime). A slight underestimation of the EQE_{EL} (≈1.25×) cannot be excluded at present as some photons from the solar cells may escaped to the side and were not detected. A forward bias was applied to the cell using a Keithley 2400 source-meter and the injected current was monitored. Measurements were conducted with a home written LabVIEW routine. Typically, the voltage was increased in steps of 50 mV and the current stabilized for typical 1s at each step. No relevant changes in the EQE_{EL} were observed for different stabilization times.

RPV Measurements: Photovoltage transients were recorded with an Agilent 81150A oscilloscope using an external load resistances (R_{Load}) of 1 MΩ.[5] A Q-switched neodymium-doped yttrium aluminum garnet (Nd:YAG) laser (NT242, EKSPLA)) with a pulse length of 5 ns and excitation wavelength of 545 nm was used to generate the charge carriers, while neutral optical density (OD) filters were used to attenuate the power output. The laser fluence was varied (resulting in a photovoltages between 50 mV to 400mV at an R_{Load} of 1 MΩ) but kept significantly below the open-curcuit voltage to avoid significant field screening effects. The measurements were performed at a repetition rate of 10 Hz and averaged to extract low-noisy measurements.

External quantum efficiencies (EQE): External quantum efficiencies were measured using a Bentham PVE system using a chopped monochromated light source, a pre-amplifyer and a lock-in-amplifyer. For sensitive EQE measurements with a signal to noise ratio above 46 dB the bandwith was decreased following ref. [6]

Photoluminescence Lifetime Imaging and transient Photoluminescence: For photoluminescence lifetimes mapping we employed a confocal single-photon counting fluorescence microscope from Picoquant. We used an excitation of λ = 636 nm through a 100x long working distance air objective (NA = 0.8), and collected the photoluminescence through a dichroic mirror, a λ = 640 nm long-pass filter, and a 50 µm pinhole. The excitation and emission were then raster-scanned using a galvo mirror system, where both the objective and sample remain at a fixed position. Excitation densities and repetition rates, are given in the main text in the corresponding figure captions.

References

1. Jacobsson, T.J., Hultqvist, A., García-Fernández, A., Anand, A., Al-Ashouri, A., Hagfeldt, A., Crovetto, A., Abate, A., Ricciardulli, A.G., Vijayan, A., et al. (2021). An open-access database and analysis tool for perovskite solar cells based on the FAIR data principles. Nat. Energy.

2. Chiang, Y.-H., Anaya, M., and Stranks, S.D. (2020). Multisource Vacuum Deposition of Methylammonium-Free Perovskite Solar Cells. ACS Energy Lett. *5*, 2498–2504.

3. Denker, A., Rethfeldt, C., Röhrich, J., Berlin, H., Cordini, D., Heufelder, J., Stark, R., Weber, A., and Berlin, B.H. (2010). Status of the Hzb # Cyclotron : Eye Tumour Therapy in Berlin Patient Numbers and Special. Proc. CYCLOTRONS 2010, (Lanzhou, China), 75–77.

4. Röhrich, J., Damerow, T., Hahn, W., Müller, U., Reinholz, U., and Denker, A. (2012). A Tandetron™ as proton injector for the eye tumor therapy in Berlin. Rev. Sci. Instrum. *83*, 02B903.

5. Philippa, B., Stolterfoht, M., Burn, P.L., Juška, G., Meredith, P., White, R.D., and Pivrikas, A. (2014). The impact of hot charge carrier mobility on photocurrent losses in polymer-based solar cells. Sci. Rep. *4*, 5695.

6. Zeiske, S., Kaiser, C., Meredith, P., and Armin, A. (2020). Sensitivity of Sub-Bandgap External Quantum Efficiency Measurements of Solar Cells under Electrical and Light Bias. ACS Photonics *7*, 256–264.

7. American Society for Testing and Materials (ASTM) Terrestrial Reference Spectra for Photovoltaic Performance Evaluation (2017). Reference Solar Spectral Irradiance Air Mass 1.5 ASTM G173. http://rredc.nrel.gov/solar/spectra/am1.5/.
